# Long-term monitoring of *Ziphius cavirostris* behavior using 3D tracking from fixed hydrophone arrays off Southern California

**DOI:** 10.1038/s41598-025-24490-x

**Published:** 2025-11-19

**Authors:** Lauren M. Baggett, Eric R. Snyder, Alba Solsona-Berga, Isabelle J. Curran, Sean M. Wiggins, John A. Hildebrand, Ana Širović, Kaitlin E. Frasier, Simone Baumann-Pickering

**Affiliations:** 1 Scripps Institution of Oceanography, University of California San Diego, La Jolla, CA 92093 USA; 2 Department of Biology, Norwegian University of Science and Technology, Trondheim, Norway; 3 K. Lisa Yang Center for Conservation Bioacoustics, Cornell Lab of Ornithology, Cornell University, Ithaca, NY 14850 USA

**Keywords:** *Ziphius cavirostris*, Cuvier’s beaked whale, Bioacoustics, Localization, Foraging, Diving, Ocean sciences, Ecology, Behavioural ecology, Conservation biology

## Abstract

Goose-beaked whales (*Ziphius cavirostris*) are a deep-diving toothed whale species and top predators in deep sea ecosystems. Much is yet to be learned about their social and foraging strategies due to their elusive behavior, but this information is increasingly relevant given their demonstrated behavioral changes in association with anthropogenic sound. This study used direction-of-arrival (DOA) localization to track the position of goose-beaked whales from echolocation clicks recorded on seafloor-mounted hydrophone arrays offshore Southern California. Overall, 2738 tracks of diving goose-beaked whales were processed from acoustic recordings collected at four long-term monitoring sites between 2018 and 2023. Results highlight distinct spatial use patterns driven by bathymetric features at each site, with whales foraging closer to the seafloor at sites with complex bathymetry and showing a preference for certain bathymetric features. Group sizes at depth ranged from 1 to 9 individuals with a mean of 2.34 and exhibited site-specific seasonal variability as well as a strong diel trend at one site. During many of these encounters, individuals exhibited highly coordinated behaviors. This study demonstrates the value of long-term passive acoustic tracking for studying elusive, deep-diving species and provides significant advancements in understanding goose-beaked whale behavior at depth over long time scales.

## Introduction

*Ziphius cavirostris*, commonly known as goose-beaked whales or formerly Cuvier’s beaked whales^1^ are an odontocete species belonging to the beaked whale family *Ziphiidae*^2^ and distributed throughout global tropical and temperate oceans^3^. Beaked whales as a group are known for their extreme diving behavior^4^ with goose-beaked whales holding both current mammalian dive depth and duration records (2992 m, 137.5 m)^5^.

Like other odontocete species, goose-beaked whales are highly soniferous and produce echolocation clicks with species-specific characteristics to navigate and forage in the darkness of such extreme depths^6,7^. Stomach content analyses from stranded individuals indicate that they are top predators in deep-sea ecosystems; their primary prey are deep-sea cephalopods with occasional mesopelagic fish and crustaceans^8–10^. Understanding and monitoring goose-beaked whale social and foraging behavior at depth can provide insights that will support management efforts, effective protection strategies, and accurate impact assessments.

Studies using instrumented tags have provided insight into the common diving patterns of goose-beaked whales. They regularly perform acoustically active deep dives to the mesopelagic and bathypelagic zones, lasting about one hour^4,11–14^. These deep dives are the only ones during which whales produce echolocation clicks and buzzes, indicating they are primarily for the purpose of foraging^11–15^. These deep dives are interspersed with silent surface intervals of variable durations ranging from a few minutes to over an hour as well as short, shallow dives that have not been observed to include foraging^4,5,11,13,14^ and serve an unclear purpose^16,17^. These tag data provide insight into the full range of diving patterns of individual cetaceans, but it is exceedingly rare to tag more than a single individual within a group simultaneously^18^. These data are needed to understand goose-beaked whale collective behavior.

Passive acoustic time-difference-of-arrival (TDOA) localization methods provide an alternate approach to tracking cetacean behavior that might yield more information on group behavior at depth. These methods have previously been deployed on short-term timescales ranging from hours^19^ to weeks^20^ to study goose-beaked whales. Data from longer-term deployments on the order of months have been used for equipment and algorithm development^21–23^ rather than complete analysis of large numbers of encounters. We have taken the next step in processing long-term monitoring datasets using these techniques to provide insights related to fine-scale presumed foraging behaviors, spatial interactions, and conspecific interactions between goose-beaked whales at depth and over long time scales.

The Southern California Bight (SCB), which includes the water offshore Southern California between San Diego and Point Conception, is a known goose-beaked whale habitat featuring a network of basins and submarine canyons^24,25^. The oceanography of this region is defined as an Eastern Boundary Upwelling System and is shaped by the southward-flowing California Current carrying cool, nutrient-rich water^26,27^, and the northward-flowing California Undercurrent transporting warmer, nutrient-rich water^26,28^. Water mass distributions are influenced by seasonal and interannual variability in these currents, especially during El Niño-Southern Oscillation (ENSO) events, which in turn influence productivity and impact the habitat of top predators like goose-beaked whales^29^. This stretch of coastal ocean is also of economic, military, and ecological interest: bisected by commercial shipping lanes, the SCB includes the Channel Islands National Marine Sanctuary and an offshore naval range used for regular training exercises^24,25^. Goose-beaked whales have exhibited behavioral changes including stranding in association with naval mid-frequency active sonar^30–33^. The exact mechanisms behind these behavioral changes remain unclear but have been the subject of considerable research effort^30,31,33–35^. Understanding baseline diving behaviors will inform effective population management strategies and anthropogenic noise risk assessment.

In this study, we constructed 2738 three-dimensional (3D) tracks from goose-beaked whale deep dives using echolocation clicks recorded on a pair of seafloor-mounted small-aperture 4-hydrophone arrays at four sites within the SCB between 2018 and 2023. Dives were tracked by computing TDOAs of individual echolocation clicks to provide the bearing angles from each array which were then cross-fixed to estimate whale positions^21,23^. This approach provided monitoring of goose-beaked whales at specific locations of interest and enabled detailed analysis of individuals and groups echolocating over an extended time period. From this dataset, we described dive phases and swim speeds, then characterized dive behavior in relation to bathymetry, group sizes, and group behaviors, and compared these patterns across sites and over time. Insights from this dataset revealed previously unknown goose-beaked whale behaviors at depth and showed that diving behavior can be studied long-term and across many echolocating individuals using this passive acoustic tracking approach.

**Fig. 1:**
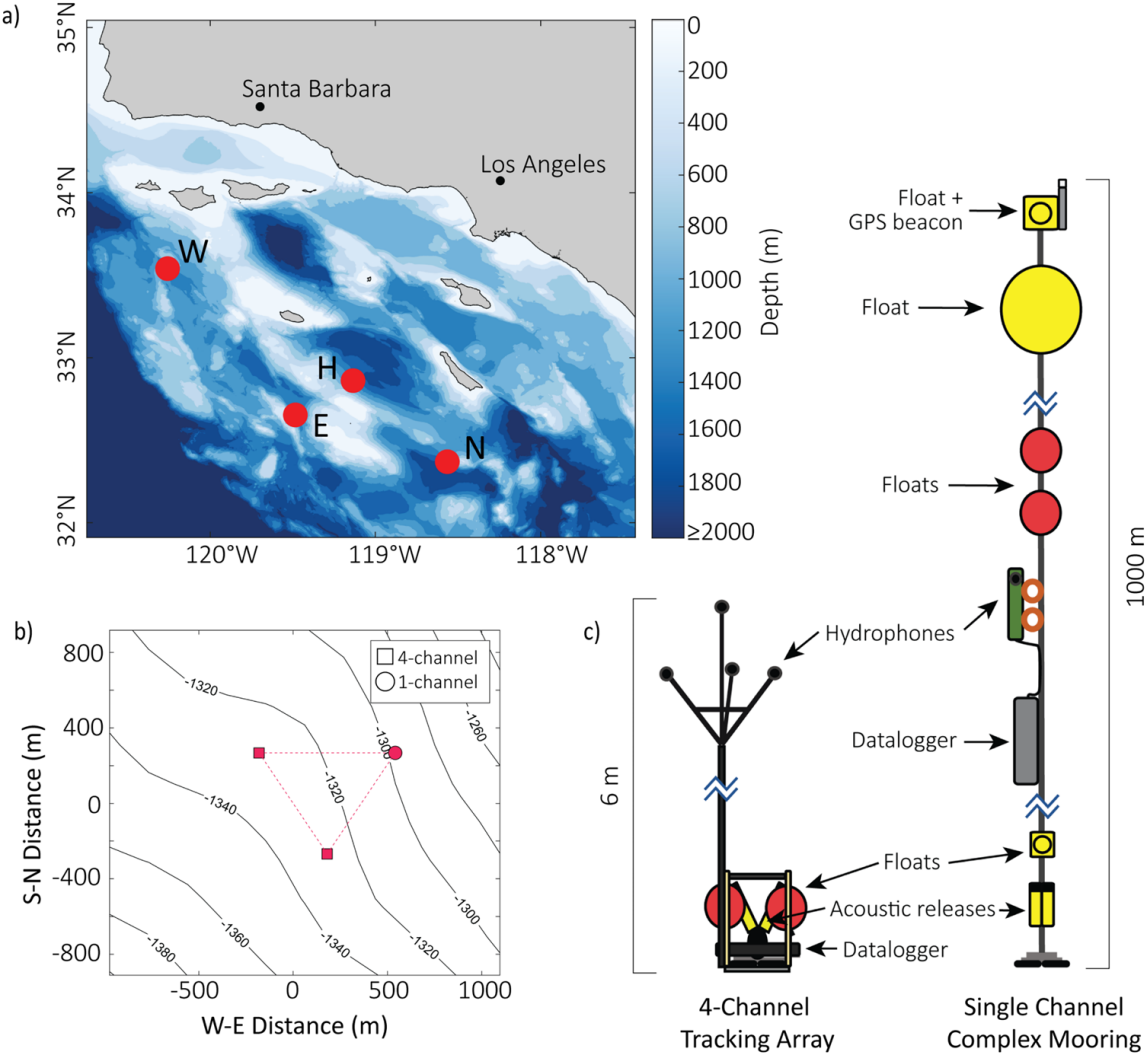
(**a**) Map of study area. Red dots represent HARP deployment sites. Bathymetry data are from GMRT (https://www.gmrt.org/)^36^. (**b**) Instrument deployment configuration. One deployment of three passive acoustic recorders from site N shown as an example. Square markers represent 4-hydrophone small-aperture arrays, and circle marker represents the single hydrophone mooring. c) Diagram of instrumentation. Renderings of a 4-hydrophone array and a single hydrophone recorder.

## Results

### Goose-beaked whale acoustic presence

**Fig. 2:**
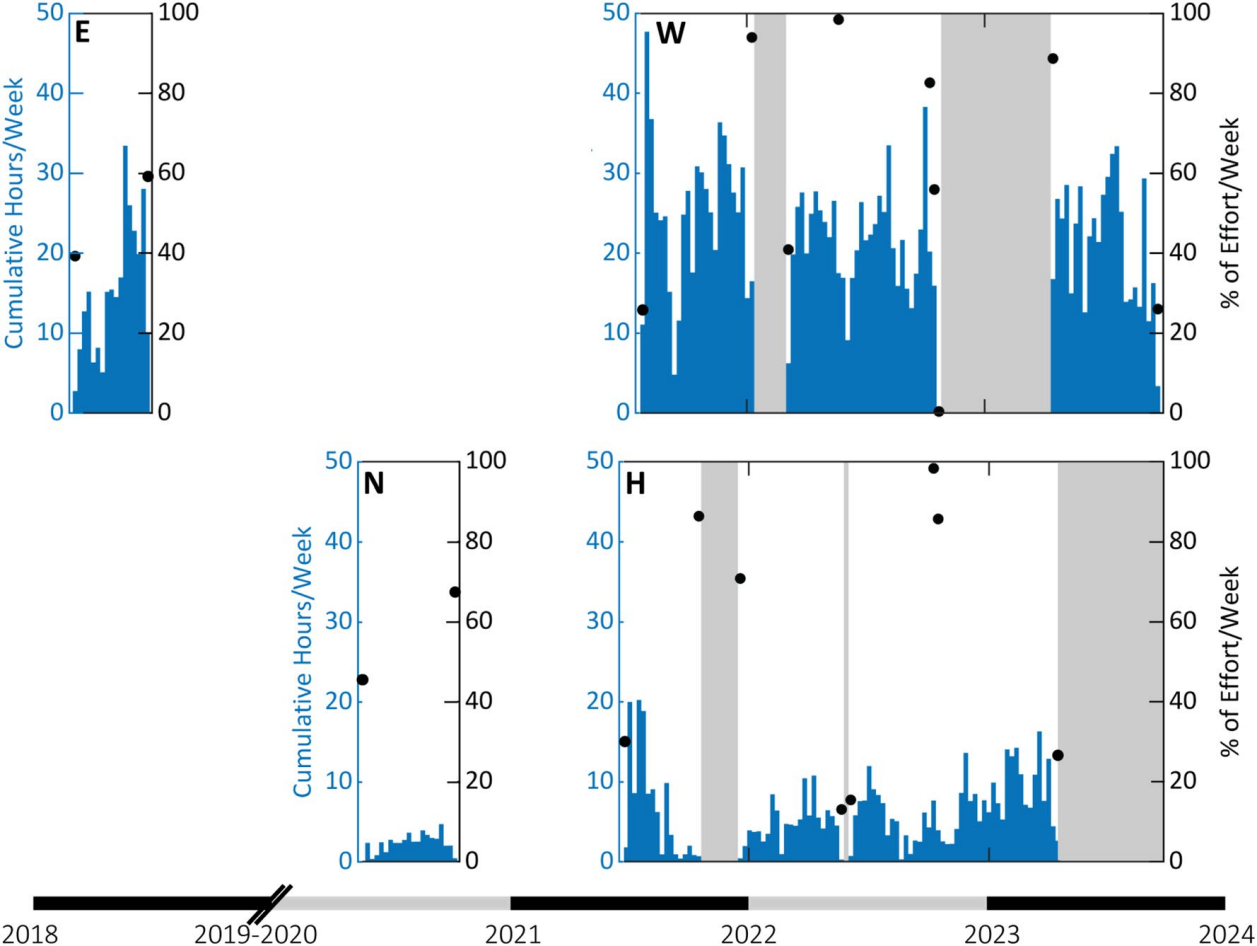
Goose-beaked whale acoustic presence from detection of echolocation clicks on single hydrophones at each monitoring site during periods when tracking arrays recorded. Black dots represent percent effort in weeks with less than 100% recording efforts, and gray shading represents periods with no recording effort. Site letter is indicated on each time series. Diagonal black lines across the x-axis indicate a gap in recording effort for 2019.

Goose-beaked whale echolocation clicks were detected at all four study sites in the SCB and during all deployments (Fig. 1). Site W had the highest acoustic presence (99.5% of recorded days were click-positive for goose-beaked whales, n = 553.9 days), followed by site E (97.3% click-positive days, n = 111.9 days), site H (80.7% click-positive days, n = 575.9 days), and site N with the lowest (56.2% click-positive days, n = 140.9 days) (Table 1; Supplemental Table 1; Fig. 2).

### Goose-beaked whale tracks

A total of 2738 tracks of diving goose-beaked whales were processed from 1187 encounters (Supplemental Figs. 1; 3a; 4a). Mean track duration was 13 min 40 s, and mean encounter duration was 20 min 41 s (Supplemental Fig. 2). The longest tracked duration for an individual was 46 min 34 s. An additional 483 tracks could not be localized to a final 3D position because the whales were not recorded on both 4-hydrophone recorders simultaneously; these tracks were only included in group size analysis.

### Spatial use: vertical

**Fig. 3:**
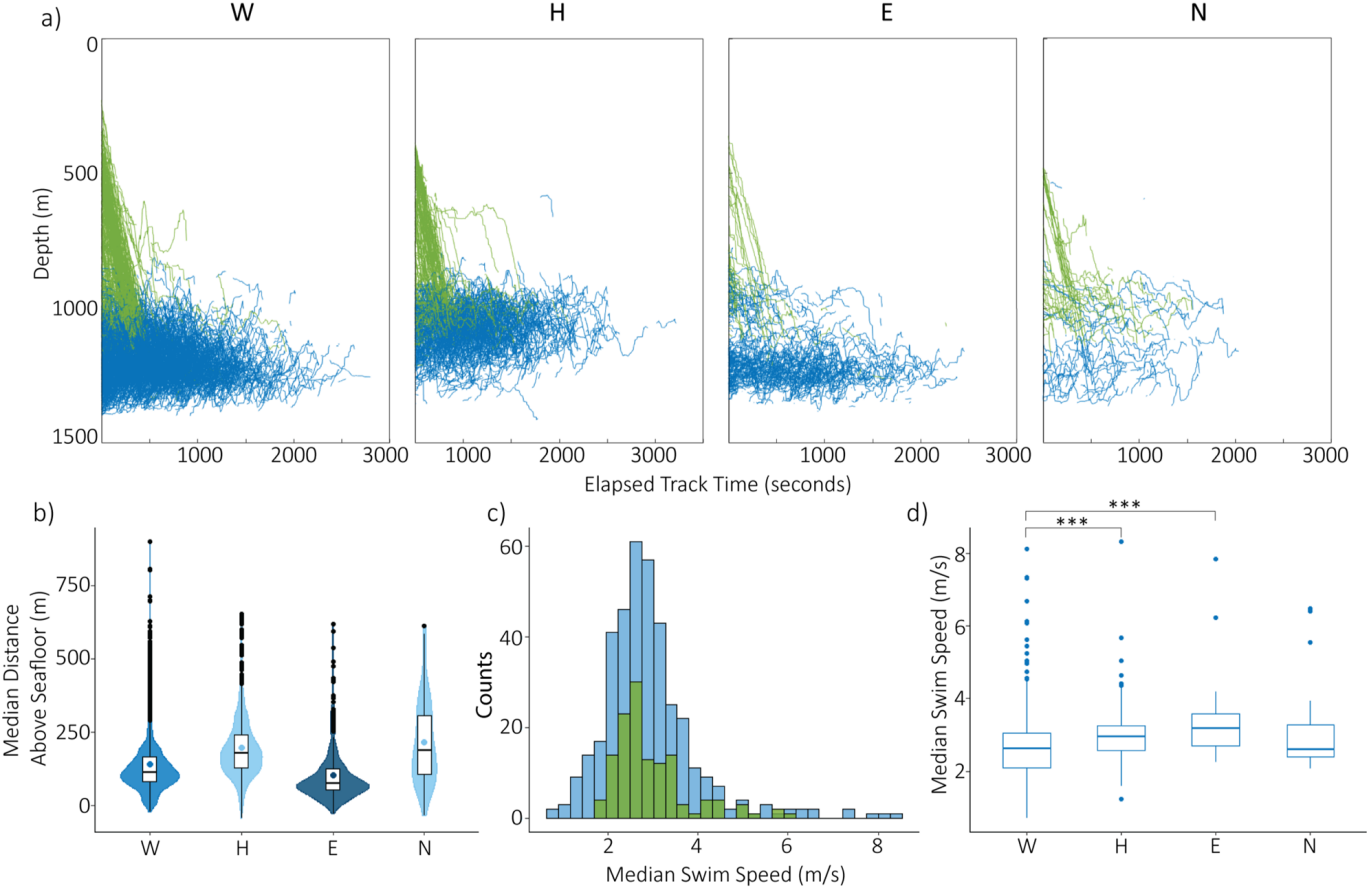
(**a**) All tracked whales per site, depth vs time. Green coloring represents the initial descent phase and blue coloring represents the at depth phase. Four panels represent the four sites. (**b**) Box and violin plot of median distances above the seafloor for whales in the foraging portion of their dive (blue) per site. Shading (dark, medium, light) represents groups that are significantly different to each other. (**c**) Distribution of median swim speeds for all sites. Green coloring represents median swim speeds for initial descent and blue coloring represents at depth phases. Swim speeds between these phases were not significantly different. (**d**) Boxplots showing median swim speeds at depth (blue) per site. Significantly different pairs are indicated with a bracket and adjusted *p* value significance denoted as ***p < 0.001.

To determine which portions of a deep dive were captured in this analysis, we looked at the depth distributions of the tracked whales on “initial descent” (from surface to 200 m above seafloor) and “at depth” (time spent within 200 m of the seafloor) phases (Fig. 3a). The angle of descent during the initial descent dive phase was −68.8$^{\circ }\pm 12.3^{\circ }$ relative to the sea surface.

Distinct vertical spatial use trends were observed between sites for whales at depth (Fig. 3b). Median distances above the seafloor were significantly different between sites (Kruskal-Wallis test, p < 0.001). Site E was significantly different from all other sites and had the lowest median distance above the seafloor at 74 m. Site W was significantly different from all other sites and had the second lowest median distance above the seafloor at 115 m. Sites H and N were statistically different from sites W and E but not to each other and had median distances above the seafloor of 179 m and 189 m respectively (Supplemental Table 2).

Initial descent median swim speeds were not significantly different from swim speeds at depth (Kruskal-Wallis, p = 0.676) (Fig. 3c). Some significant differences in median swim speeds while whales were at depth were observed between sites (Kruskal–Wallis, *p* < 0.001); site W was significantly different from sites H and E (Fig. 3d; Supplemental Table 3).

### Spatial use: horizontal

**Fig. 4:**
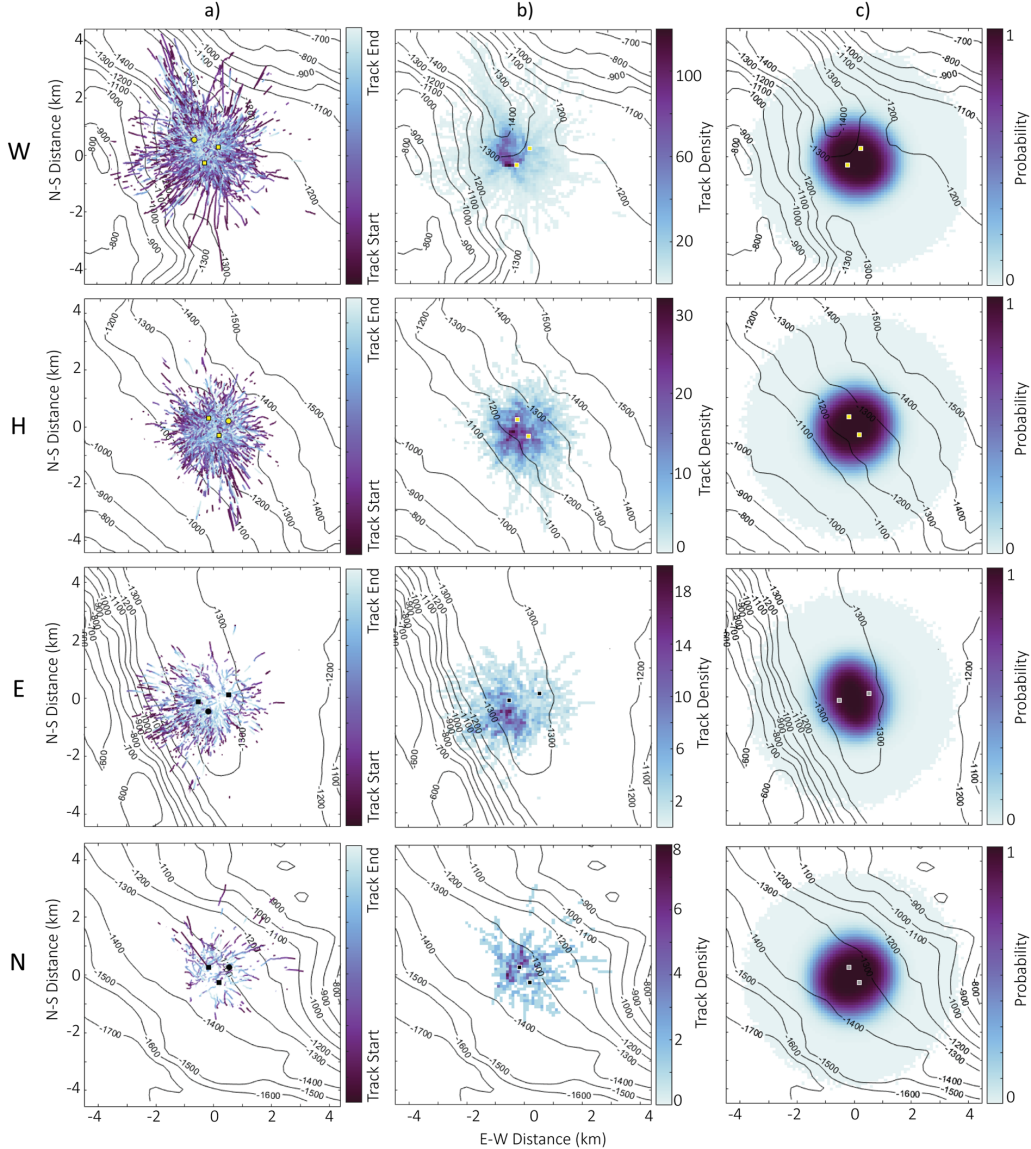
(**a**) Tracked whales by site. Tracks are colored by normalized track time, with the start of the track in purple and the end of the track in light blue. Square markers represent 4-hydrophone arrays, and circle markers represent single hydrophones. Black markers are used at sites with one deployment period and yellow markers are used at sites with multiple deployments, delineating the position of the first deployment. Bathymetric contour lines are every 100 m. (**b**) Densities of tracked whales per site shown using 10 m by 10 m grid squares colored by the number of tracked whales within the squares. (**c**) Probabilities of tracking an echolocating whale due to hydrophone array geometry based on Monte-Carlo simulation, normalized between 0 and 1.

Tracks were visualized in the XY plane to identify horizontal spatial trends. At all sites, tracks were best obtained when the whales were swimming from all directions toward the 4-hydrophone instruments (Fig. 4a). This was an outcome of the directionality of goose-beaked whale echolocation clicks, which are higher amplitude in the rostral direction^6^. At site W, the area with the highest track density was just to the north of the 4-hydrophone instruments in a narrow, deeper channel where the seafloor slopes downward (Fig. 4b). At site E, the area with the highest track density was southeast of the 4-hydrophone instruments near a canyon slope (Fig. 4b). At sites H and N, the areas with the highest track densities were evenly distributed closely around the 4-hydrophone arrays (Fig. 4b).

At all sites, a “hotspot” of high tracking probability appeared in an ellipse around the two 4-hydrophone arrays with a steep dropoff in probabilities for another several km (Fig. 4c). The elliptically-shaped hotspot of tracking probabilities aligned well with the observed areas of highest track density at sites H and N, but did not match the areas of highest track density at sites W and E (Fig. 4b; 4c), suggesting that the bathymetric features at these two sites may influence foraging.

### Group size

Goose-beaked whales were observed either solitary or in groups ranging from 2 to 9 individuals (median 2 whales). To evaluate whether group size varied temporally, with either a diel or seasonal pattern, Generalized Additive Models (GAMs) were constructed. The best model at site W included Julian day and goose-beaked whale acoustic presence as smooth terms (adjusted $\textrm{R}^2$ = 0.083, deviance explained = 9.77%; Supplemental Fig. 6a). The best model at site H included Julian day and normalized time of day as smooth terms, and goose-beaked whale acoustic presence as a linear term (adjusted $\textrm{R}^2$ = 0.077, deviance explained = 10.1%; Supplemental Fig. 6b).

**Fig. 5:**
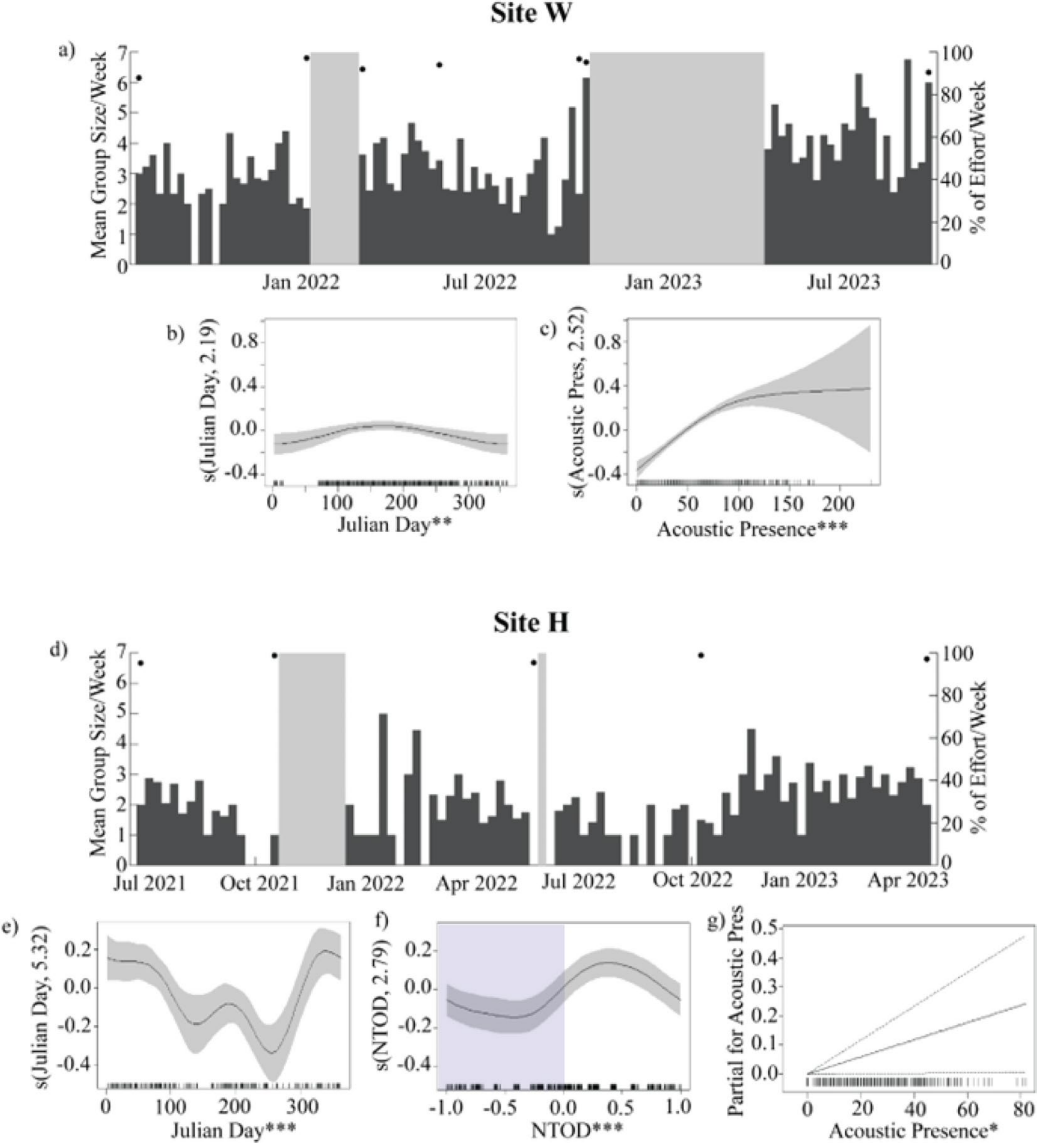
Time series showing mean group size per week at site W (**a**) and H (**d**). Black dots represent percent effort in weeks with less than 100% recording effort, and gray shading represents periods with no recording effort. Generalized Additive Model (GAM) results showing relationships between the probability of goose-beaked whale group sizes and Julian day (**b**) and (**e**), normalized time of day (NTOD, sunrise at 0 and sunset at −1/1 with blue shading representing nighttime, (**f**), and acoustic presence (**c**) and (**g**) with group size. GAMs are fit separately for each site, and not all predictors were retained during model selection for all sites. Rug plots show distribution of predictor variables. *p* value significance is denoted by ***p<0.001; **p<0.01; and *p<0.05. Model fit is shown as a black line, 95% confidence intervals are shown as either a shaded area (for smooths) or a dashed line (for factors).

Seasonal trends in group size were observed at both sites W and H, although more pronounced at site H. At site W, group size peaked in the summer months (June to August) and declined in the winter months (November to February, Fig. 5b). Conversely, at site H, group size was largest in the winter months with a smaller, secondary peak during the summer (Fig. 5e). Diel patterns in group size were only significant at site H, with larger group sizes during the day (Fig. 5f); model selection at site W did not retain normalized time of day. Group sizes were higher when more click-positive minutes were recorded on the single hydrophone at site W (Fig. 5c), but the relationship between these variables was not as pronounced at site H (Fig. 5g).

### Group behaviors

**Fig. 6:**
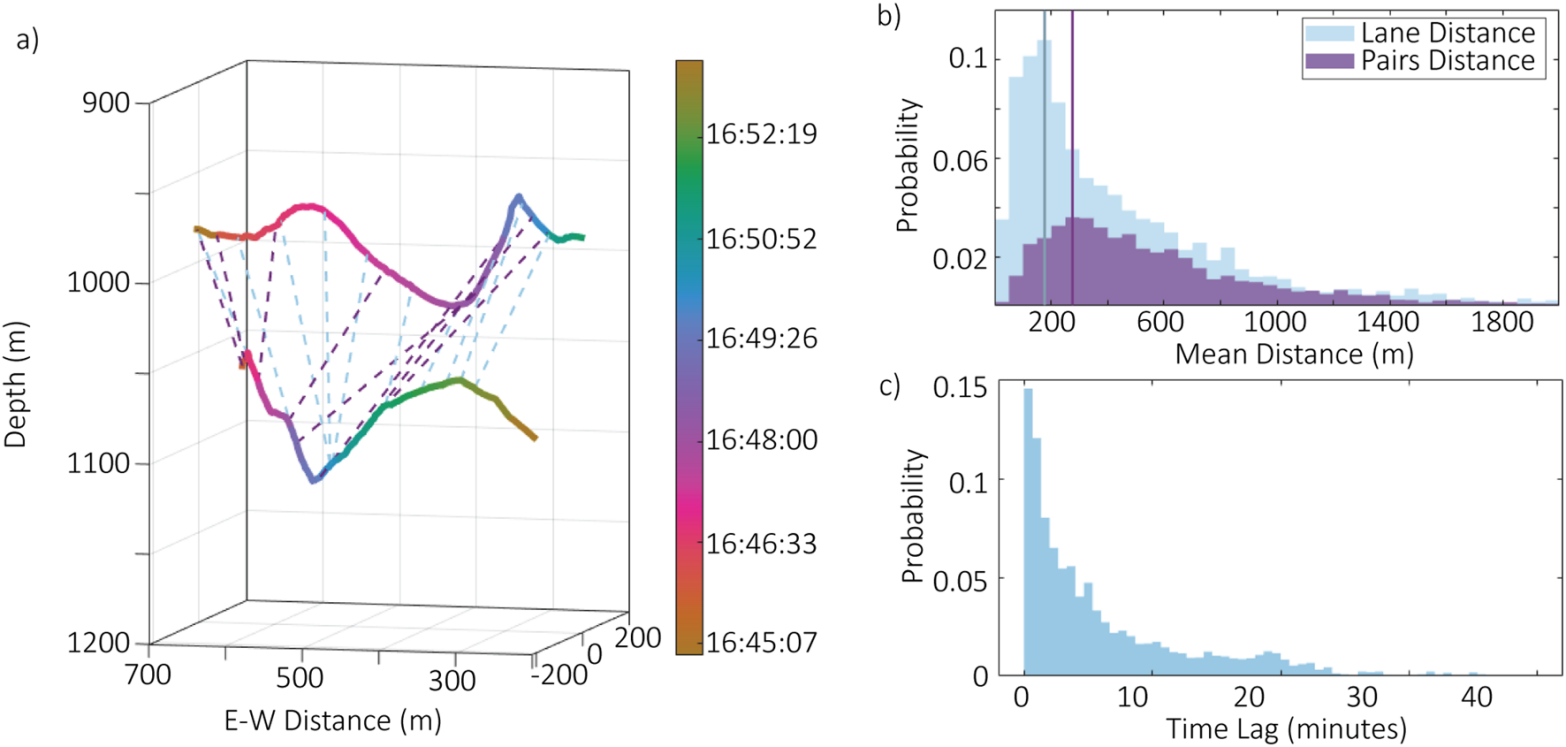
(**a**) Encounter with two whales at depth (solid lines) colored by time, illustrating distances with respect to time (pairs distance, purple dashed lines) and at closest points of approach without considering time (lane distance, blue dashed lines). For clarity, only a portion of calculated lane distances are plotted. (**b**) Distance between pairs of whales in an encounter with respect to time (pairs distance, purple) and without respect to time (lane distance, blue) at all sites. Lines represent modal bins for each. (**c**) Histogram of time lags between the closest points of approach for whale pairs per encounter at all sites.

Individuals often exhibited coordinated behaviors (see animations in Supplement). During these encounters, whales traveled similar trajectories but were slightly offset in both space and time (Fig. 6a). Most whales remained within 1 km of each other with a modal distance between 250 and 300 m (pairs distance, Fig. 6b). When ignoring time and considering only spatial proximity (lane distance), their paths were even closer with a modal lane distance between 150 and 200 m (Fig. 6b). The modal time lag between the closest recorded points in space for two whales was less than one minute (Fig. 6c).

**Fig. 7:**
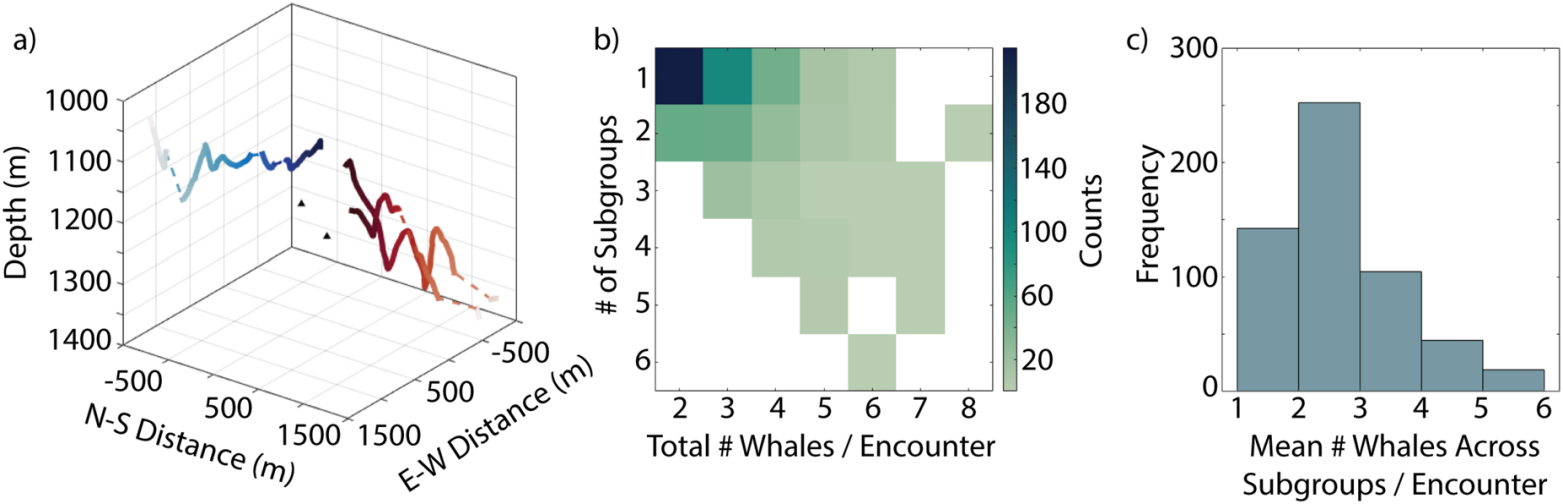
(**a**) Encounter with three whales forming two subgroups indicated by color (red and blue). Shading represents time, from light (start of track) to dark (end of track). Gaps in detections for an individual are represented as a dashed line. Black triangles denote positions of 4-hydrophone receivers. (**b**) Number of subgroups per encounter for all deployments by total number of whales present, color represents counts. (**c**) Distribution of mean number of whales across subgroups per encounter.

In encounters involving larger numbers of individuals, the whales sometimes exhibited a subgrouping behavior where certain individuals appeared more closely associated with each other rather than being equally associated with all whales in the encounter (Fig. 7a). Of the 563 encounters that included more than one whale, 184 of them exhibited some level of subgrouping. As the total number of whales recorded in an encounter increased, the number of subgroups per encounter also increased (Fig. 7b). These subgroups consisted of 1 to 6 whales with a mean of 2.27 (Fig. 7c).

## Discussion

These results demonstrate that passive acoustic tracking is an effective method for studying goose-beaked whale deep dives at fixed locations. This analysis yielded 2738 tracks of portions of goose-beaked whale deep dives from over three years of acoustic data collected at four sites. Diving behavior differed between sites, with tracks recorded closer to the seafloor and in higher densities near bathymetric features at sites W and E. Temporal variations in group size, both seasonal and diel, were observed at site H. At all sites, whales exhibited coordinated behaviors and subgrouping at depth. These tracks provide insight into the presumed foraging behaviors of this species on fine-scale spatial and temporal resolutions, aggregating patterns on long-term timescales.

These tracks represent portions of goose-beaked whale deep dives, which are the only dives for this species to typically include echolocation clicks and buzzing indicative of active foraging^4,11–15^. Given the significantly lower source level of buzzes^6^, they are infrequently recorded in our data and we are unable to directly observe prey capture attempts; we presume these tracks represent foraging behavior because they capture echolocation at depth and align well with active foraging behaviors consistently exhibited by tagged whales^4,11–14^.

Differences in track median swim speeds and distances above the seafloor were observed between sites. Whales also exhibited a spatial preference for distinct bathymetric features, favoring a canyon wall (site E) and a narrow, deeper channel of a submarine canyon (site W), as indicated by track densities relative to detection probabilities. Since these tracks likely represent foraging, these differences in swim speeds and behavior in relation to bathymetry may indicate differences in foraging strategies between sites. Little is known about goose-beaked whale prey distributions at depth, but some studies in this region suggest that they are variable across small horizontal scales^37^ and are linked to physical oceanographic conditions^29^. Advancing our understanding of the prey community at these sites through methods like net tows, underwater camera systems, and active acoustics would provide context needed to better interpret the fine-scale spatial use trends observed here. Active acoustic data were simultaneously collected on some of the deployments in this analysis, and we aim to combine insights from those datasets with these tracked goose-beaked whale dives in a future study.

The probability of tracking was modeled for each deployment using a Monte Carlo simulation approach modified from Hildebrand et al. 2015^38^. This method depends on several parameters with limited data for this species, particularly echolocation click directivity and source level^38^. The probability of tracking a whale was modeled for a range of values around reported numbers for click directivity and source level^6,21^. Reported values were obtained using small sample sizes, and more variability than anticipated in these parameters would increase our uncertainty in the estimate of tracking probability. One application of this dataset would be to calculate click source level and directivity using a subset of these tracks. These parameters could be used to fine-tune the probability of tracking a whale for each deployment, and further to improve other existing models estimating the population density of goose-beaked whales using acoustic detections^38,39^.

At sites with more than one year of tracking effort (W and H), GAM models suggest that group size varies with Julian day. A time series from long-term passive acoustic monitoring at site H shows higher goose-beaked whale presence during winter (November to January) and spring (March to May)^29^, however we observed larger group sizes in our analysis during the summer months (June to August) and winter (November to February). This may indicate that the spring peak observed by Schoenbeck et al. is comprised of small group sizes or individual whales. Long-term studies of goose-beaked whales using visual surveys^24,40^ and tagging^5,25^ show long-term site fidelity of some individuals to the San Nicolas Basin, near site H, indicating that this region is an important habitat for goose-beaked whales. Similar efforts have not yet been conducted near site W, but group sizes at this site were slightly higher during summer months.

Although goose-beaked whale echolocation clicks are species-specific^6,7^, our passive acoustic tracking method cannot differentiate between individuals belonging to different populations and cannot identify whether the same individual is tracked multiple times across different deep foraging dives. Furthering our understanding of goose-beaked whale residency patterns using visual sighting, tagging, and genetic methods in these monitoring regions may improve interpretation of the seasonal group size trends observed here.

Larger group sizes were observed at site H during the daytime, while no diel trend was observed at site W. One study suggests that goose-beaked whales in this region spend a greater fraction of time foraging at night^14^, although other studies found that foraging dive duration was not related to time of day^5,25^. However, goose-beaked whales appear to spend more time near the surface at night^5,14^, a behavior hypothesized to be consistent with avoidance of near-surface visual predators during the day^14^. Since goose-beaked whales do not produce echolocation clicks on their ascent, near the surface, or between deep dives^4,15^, our dataset did not capture deep dive ascents or inter-deep dive behaviors that may help contextualize our observed diel group size trend. Further understanding of goose-beaked whale diel foraging behaviors using tags, and by tagging multiple individuals within a group, would facilitate our interpretation of this finding. Additionally, a lack of a diel group size trend at site W suggests that the drivers of group size variation may differ across the SCB region.

Click-positive minutes, a commonly used metric for aggregating odontocete acoustic detections over long time periods^29,41,42^, correlated strongly with group size at site W and weakly at site H. Some degree of vocal coordination between goose-beaked whales at depth is a phenomenon which has been previously described from tag recordings in the Ligurian sea and the Azores^18,43^, although based on a small sample size of simultaneously tagged whales. Aguilar de Soto et al. finds goose-beaked whales to overlap vocal foraging time as much as 98%^18^. Similarly, the weak association between click-positive minutes and group size at site H indicates that goose-beaked whales may overlap vocal foraging time at this site.

A limitation of passive acoustic tracking is that individuals can only be tracked when they are within the recording radius of the passive acoustic devices and producing echolocation clicks. More whales may be nearby and either not echolocating or positioned outside of the detection range of the sensors; it is subsequently unclear to what spatial scale these group size estimates are representative. The group sizes reported here should therefore be interpreted as a minimum estimate of the number of whales foraging within the recording radius of our sensors per encounter.

Many of the encounters processed in this analysis show whales that appear highly associated with each other in space and time while conducting deep dives. Although existing information about sub-surface group behavior is limited, individual click and buzz rates suggest that whales diving in groups forage separately and do not rely on echoes from conspecifics^43^. Our findings show that the modal distance between pairs of whales at depth was less than 300 m, and that subgroups consisting of two to three very closely associated whales sometimes formed during encounters made of large group sizes. Although these distances may still represent whales which are swimming independently of one another, given that they often exhibit parallel movements within this range or travel toward other individuals at depth, we hypothesize that at least some of these swims are coordinated. Understanding of whale age, sex, and size would be informative and may reveal clearer patterns, but this information cannot be determined acoustically for goose-beaked whales. We also cannot determine if these same individuals remained closely associated at the surface or across multiple deep dives.

This passive acoustic tracking method enabled short-term tracking of individuals or groups, giving a snapshot view of their behaviors while conducting deep dives. Continuous tracking effort over long-term time scales can help identify fine-scale spatial and group behaviors that persist at specific monitoring locations, but this method does not include information about the broader range of diving behaviors a whale exhibits while not echolocating. Many of our findings vary between sites, and it is unclear which site-specific results are representative of broader spatial patterns. Goose-beaked whale foraging behavior may also be influenced by factors like oceanographic conditions, prey distributions, or anthropogenic noise and future analyses should explore these possibilities.

## Conclusions

A total of 2738 tracks of diving goose-beaked whales were recorded using hydrophone arrays at four sites in the Southern California Bight. The results demonstrate that passive acoustic tracking is an effective method to study presumed foraging and social behavior of deep-diving odontocetes in an area of interest over long timescales. These findings reveal that goose-beaked whales exhibited fine-scale behavioral variability. Understanding these behaviors at depth may be key to developing management strategies for goose-beaked whale populations.

## Methods

### Data collection

Long-term, high-frequency acoustic recordings were collected at four sites offshore Southern California (Fig. 1a). Site W is located south of Santa Rosa Island, in a bowl-shaped edge of a submarine canyon. A narrow, deeper channel of this canyon lies just to the north of the instrument locations. Site H is west of San Clemente Island on the slope of the San Nicholas Basin, though the immediate bathymetry at this site is gently sloping. Site E is west of San Clemente Island on the eastern-facing slope of Tanner Basin with a steep slope just to the west of the instruments. Site N is south of San Clemente Island on the western-facing slope of the East Cortes Basin, and the immediate bathymetry around this site is gently sloping. All sites are between 1200 and 1350 m deep (Fig. 1; Supplemental Table 1; Supplemental Fig. 3). High-frequency Acoustic Recording Packages (HARPs)^44,45^ deployed at these sites continuously recorded during several multi-month deployments between 2018 and 2023 (Table 1; Supplemental Table 1). Three HARPs per deployment were positioned approximately 1 km apart and recorded simultaneously (Fig. 1b; Supplemental Fig. 3). Two of the instruments were equipped with a small-aperture array of four hydrophones sampling continuously at 100 kHz. The four hydrophones were mounted tetrahedrally approximately 6 m above the seafloor with individual hydrophones separated by 1 m (Fig. 1c). The third package was equipped with a single hydrophone sampling continuously at 200 kHz. This hydrophone was part of a multi-instrumented mooring 1000 m long. The hydrophone was attached near the bottom of the long mooring, positioned approximately 15 m above the seafloor (Fig. 1c). Locations of the 4-hydrophone arrays were calculated using the TDOAs of the ship engine noise at a variety of GPS locations and inverting for the receiver positions that best minimized the error between expected and measured TDOAs^23^. Sound speed profiles were obtained from CTD casts taken during each deployment cruise near the drop location.

**Table 1:** Summary of passive acoustic tracking effort at Southern California sites. Effort was not always continuous from start effort to end effort dates; see also Supplemental Table 1.

Site	Latitude	Longitude	Start effort	End effort	Days effort
W	33° 32.25′ N	120° 14.95′ W	Jul 30, 2021	Sept 25, 2023	553.91
H	32° 51.67′ N	119° 08.11′ W	Jul 02, 2021	Apr 17, 2023	575.85
E	32° 39.36′ N	119° 29.26′ W	Mar 14, 2018	Jul 11, 2018	111.89
N	32° 22.18′ N	118° 33.93′ W	Apr 30, 2020	Oct 11, 2020	140.91

### Identifying goose-beaked whale echolocation clicks

A machine learning workflow for detecting and classifying odontocete echolocation clicks^46,47^ was applied to the single hydrophone recordings to identify goose-beaked whale echolocation clicks. Single hydrophones were selected for this task due to their faster sampling rate (200 kHz) that captured the higher frequencies in a goose-beaked whale echolocation click^6,7^ and allowed for more confident identification.

Goose-beaked whale echolocation clicks were detected and classified using the MATLAB^48^-based software *Triton*^49^. A customized energy detector^47^ applied a five-pole Butterworth bandpass filter with edges at 5 kHz and 95 kHz and extracted signals with peak-to-peak received level $$\ge 118\ \text {dB}\ \text {re}\ 1\\{ \mu} \text {Pa}^2$$ and durations 30 to 1200 μ$\text {s}$. A two-phase unsupervised clustering algorithm identified and grouped recurring signals based on spectra^46,47^. These clusters were manually labeled and used to train a neural network^47^. The neural network was trained to recognize biological signals (echolocation clicks from *Lagenorhynchus obliquidens*^50^, *Grampus griseus*^50^, *Ziphius cavirostris*^6,51^, and presumed *Tursiops truncatus*, *Delphinus capensis*, and *Delphinus delphis*) as well as anthropogenic signals (boats, echosounders) commonly found in data from this region. The neural network labels were manually verified in the MATLAB-based software *DetEdit*^52^. This workflow culminated with the successful identification of times when goose-beaked whale echolocation clicks were recorded on the single hydrophone system. The whale tracking methods were then applied to the 4-hydrophone data at times when goose-beaked whale clicks were present on the single-hydrophone recorder.

### Localization

Diving whales were localized using the MATLAB-based software package *Where’s Whaledo*^23^. The 4-hydrophone data, at only the detection times from the single hydrophone data, was high-pass filtered with a cutoff frequency of 20 kHz to detect click candidates with a peak-to-peak received level $\ge 112\ \text {dB}\ \text {re}\ 1\ \rm{\mu} \text {Pa}^2$. The acoustic data from each 4-hydrophone channel in a 50 $\mu \text {s}$ window around the click candidate were cross-correlated, and the time-delay between each hydrophone pair was used to estimate direction-of-arrival (DOA)^23^. These DOAs were represented as azimuth and elevation angles and plotted on the *brushDOA* interface included in *Where’s Whaledo*^23^. All tracks were manually inspected, sorted, and cleaned (Supplemental Fig. 4). Tracks with clear goose-beaked whale detections were retained and goose-beaked whale individuals were manually assigned a unique color and associated between arrays by cross-correlating echolocation click trains^23,53^. 3D whale positions relative to the receivers were calculated from the intersect of the cleaned DOAs from both 4-hydrophone arrays. The confidence of each localization was calculated using jackknifing cross-validation, wherein the position of the whale for a given detection was estimated using a subset of five of the six available TDOA pairs, and the variance of the resulting locations were used to estimate in the Student-T distribution to calculate the 95% confidence intervals^23,54,55^. To smooth the localized points, a Kalman filter^56,57^ was used to estimate position by incorporating the error of the measurements and the known correlations in the data. A moving average was applied to these estimated locations to further smooth them (Supplemental Fig. 5).

### Diving behavior classification

Whales were tracked during their deep dives, both during the descent portion (“initial descent phase”) and during presumed foraging at depth (“at depth phase”). Bathymetry data were obtained from the General Bathymetric Chart of the Oceans (GEBCO) (GEBCO Compilation Group, 2024), and at each point whale distance above the seafloor was calculated. The whale was considered to be performing its initial descent from the surface until it reached within 200 m of the seafloor, at which point it was considered to be within the presumed foraging portion of the dive. This threshold was chosen based on a study by Barlow et al. 2020, which found that *Ziphius cavirostris* in the region spent a significant portion of time foraging within 200 m of the seafloor^14^; this delineation captured the observed track behaviors well upon visual inspection (Fig. 3a).

Initial descent angles were calculated by finding the descent angle between each successive point during the initial descent phase. A kernel smoothing function estimated the probability density of the resulting distribution, and the peak of this function was reported here as the angle of descent. A standard deviation was calculated for values within 20° of this peak.

### Swim speeds

Swim speeds were estimated for each whale in each individual track using the smoothed whale positions. Distance between the 3D smoothed points was calculated using the Euclidean distance formula and then divided by the time interval between those smoothed points to get an estimate of swim speed throughout the track. Median swim speeds were calculated for both initial descent and presumed foraging portions of the dive, when both behaviors were present. For statistical analysis, only tracks with at least 5 minutes of data for initial descent phases and 10 minutes of data for at depth phases were included. Differences between initial descent and at depth phases, as well as differences in at depth speeds between sites, were analyzed by running a Kruskal-Wallis test^58^ in R version 4.4.1.^59^. Post-hoc testing using the Dunn test in the *rstatix* package^60^ in R version 4.4.1 using the Benjamini–Hochberg method to account for the multiple pair-wise comparisons identified which sites had median at depth swim speeds statistically different to each other

### Distance above the seafloor

Median distance above the seafloor for at depth portions of tracks only were considered for this analysis. Bathymetry information was obtained from GEBCO for each site. Distance above the seafloor was calculated for each track, at each time point, by finding the closest bathymetric value and subtracting the depth of the whale. After this had been calculated for each time step in the track, the median was taken. The distance above the seafloor by sites was analyzed by running a Kruskal–Wallis test in R. Post-hoc Dunn testing identified which sites had median seafloor distances that were statistically significant.

### Detection probability

The probability of single echolocation clicks being simultaneously detected on both 4-hydrophone arrays was modeled using a Monte Carlo simulation approach modified from Hildebrand et al.^38^ This approach is based on assumptions regarding source level, propagation loss, and orientation of the animals. Transmission loss was simulated using Bellhop^61^ accessed through the *PropaMod* repository (https://github.com/nposdalj/PropaMod). Bathymetry information came from GEBCO and sound speed profiles were calculated using oceanographic parameters from the ocean model HYCOM (http://hycom.org). Running one model iteration involved placing 10,000 echolocating whales within a 8 km by 8 km square area of the 4-hydrophone arrays with a random azimuth. These simulated clicks were assigned a source level and directivity value based on literature; source levels were modeled between 221 and 223 $\text {dB}\ \text {re}\ 1\ \rm{\mu} \text {Pa}^2$^21^ and directivity was modeled between 23 and 25^6^. A click was detected if the peak-to-peak received level was at or above 112 $$\text {dB}\ \text {re}\ 1\\{ \mu} \text {Pa}^2$$ (the detection threshold used in the localization steps) on both 4-hydrophone arrays. The modeled area was divided into 100 m by 100 m bins, and the detection probability for each was calculated as the number of simulated whales that were detected divided by the total number of whales simulated within that grid square. Per deployment, 135 model iterations were run (to fit a range of simulated parameters, see Supplemental Table 4) and a detection probability was calculated for each grid square. The final detection probability for each grid square was taken as the mean of these 135 model iterations. At sites with multiple deployments, detection probabilities were weighted by deployment duration for plotting (Fig. 5c).

### Group size

Group size was defined as the number of distinct individuals tracked per encounter. Detections were considered as part of the same encounter if there were no gaps in detections exceeding 30 minutes. Group size was taken as the largest number of whales recorded between the two 4-hydrophone arrays, meaning that a whale was still counted as part of the group if it was only recorded on one of the arrays and not subsequently localized to a final 3D position.

Generalized additive models (GAMs) were designed using the *mgcv* package^62^ in R to understand how changes in group size may be explained by temporal variables. Models were constructed only for sites with more than one year of data, sites W and H. Temporal predictor variables included Julian day to evaluate seasonal effects, and normalized time of day (NTOD) to assess diel variation, with NTOD scaled such that 0 represents sunrise and −1/1 represents sunset. Acoustic presence, or click-positive minutes per bin from the single-channel hydrophone, was also included as a predictor variable. The response variable, group size, was binned by summing groups into non-autocorrelated time intervals (320 minute bins at site W, 120 minute bins at site H). Autocorrelation was determined using the *acf* function in the *stats* package in R. Predictor variables were fit with the appropriate smoothing function by modeling each variable individually as a smooth or linear fit with group size and comparing Aikake Information Criterion (AIC) values. At site W, Julian day and acoustic presence were included as smooth terms. At site H, Jualian day and normalized time of day were included as smooth terms and acoustic presence was included as a linear term. Backward and forward selection was run for each model per site, and best model fits were determined as the models with the lowest AIC.

### Group behaviors

Group behaviors were first quantified by looking at the distances between individuals in time and in space. The Euclidean distance between pairs was calculated for all pair combinations of individuals that were tracked simultaneously per encounter. The mean distance was then calculated per minute for each of these pair combinations. To look at the distance between individuals independently of time, another metric referred to as “lane distance” because of its similarity to lanes on a track was calculated (Fig. 6a). Lane distance was calculated for each pair combination of whales tracked in the same deployment. The smoothed whale positions were interpolated to 1 m bins using the function *interparc*^63^ in MATLAB. For each point in one whale of the pair combination, the Euclidean distance was calculated to the nearest point in the other whale, regardless of the timestamp of the point. These lane distances were averaged per 10 m bins.

During the track cleaning step, it was observed that whales sometimes exhibited a subgrouping behavior where certain individuals were more closely associated with each other rather than being equally associated with all whales in the encounter. For encounters that contained at least two whales, a hierarchical cluster tree was created using the single linkage algorithm on the unweighted average distances between whales. Clusters were then constructed from these hierarchical cluster trees using a distance cutoff threshold. Whales in the same encounter were considered as part of separate subgroups if (1) they did not temporally overlap within 10 minutes of each other and/or (2) the distance between their clusters was greater than 1000.

## Supplementary Information

Supplementary Information 1.

Supplementary Information 2.

## Data Availability

The datasets generated and analyzed during the current study are available in the DRYAD repository, DOI: 10.5061/dryad.280gb5n1r.

## Code availability

The *Where’s Whaledo*^23^ software package can be found on GitHub (https://github.com/MarineBioAcousticsRC/Wheres-Whaledo). Code related to track analysis can also be found on GitHub (https://github.com/laurenbaggett/Zc-Tracking).
