# Supplementary material for: Long-term monitoring of Ziphius cavirostris behavior using 3D tracking from fixed hydrophone arrays off Southern California
Source: Sci Rep. 2025 Nov 19;15:40859. doi: 10.1038/s41598-025-24490-x (PMC12630762; doi:10.1038/s41598-025-24490-x)
Supplement: Supplementary file 1 — Supplementary Information 1. [file 41598_2025_24490_MOESM1_ESM.pdf]

Supplemental Table 1. Recording effort summary. Recording start and end dates, as well as position and depth, for each instrument package in each tracking array. Deployment naming convention is region (SOCAL), site, deployment number, instrument position in array (E for east, W for west, S for south). 4-channel systems are denoted by an additional "C4" at the end of the deployment name.

Deployment Name	Recording Start (UTC)	Recording End (UTC)	Lat (°N)	Lon (°W)	Depth (m)
SOCAL_W_01_WE_C4	30 Jul 2021	15 Jan 2022	33°32.249'	120°14.951'	1252
SOCAL_W_01_WS_C4	30 Jul 2021	17 Jan 2022	33°31.941'	120°15.254'	1250
SOCAL_W_01_WW	29 Jul 2021	22 Jan 2022	33°32.384'	120°15.489'	1378
SOCAL_W_02_WE_C4	09 Mar 2022	27 May 2022	33°32.221'	120°14.856'	1237
SOCAL_W_02_WS_C4	10 Mar 2022	28 May 2022	33°31.883'	120°15.097'	1237
SOCAL_W_02_WW	10 Mar 2022	27 Mar 2022	33°32.445'	120°15.550'	1375
SOCAL_W_03_WE_C4	27 May 2022	14 Oct 2022	33°32.249'	120°14.855'	1242
SOCAL_W_03_WS_C4	27 May 2022	14 Oct 2022	33°31.926'	120°15.125'	1245
SOCAL_W_03_WW	27 May 2022	14 Oct 2022	33°32.470'	120°15.553'	1384
SOCAL_W_04_WE_C4	18 Oct 2022	15 Apr 2023	33°32.281'	120°15.008'	1272
SOCAL_W_04_WS_C4	19 Oct 2022	15 Apr 2023	33°31.966'	120°15.311'	1259
SOCAL_W_04_WW	17 Oct 2022	15 Apr 2023	33°32.447'	120°15.598'	1388
SOCAL_W_05_WE_C4	16 Apr 2023	24 Sept 2023	33°32.368'	120°15.068'	1301
SOCAL_W_05_WS_C4	16 Apr 2023	24 Sept 2023	33°32.073'	120°15.282'	1284
SOCAL_W_05_WW	16 Apr 2023	25 Sept 2023	33°32.451'	120°15.554'	1385
SOCAL_H_72_HE	06 Jun 2021	19 Dec 2021	32°51.670'	119°8.110'	1283
SOCAL_H_72_HS_C4	01 Jul 2021	18 Dec 2021	32°51.391'	119°8.328'	1249
SOCAL_H_72_HW_C4	01 Jul 2021	23 Oct 2021	32°51.727'	119°8.556'	1263
SOCAL_H_73_HE	21 Dec 2021	23 May 2022	32°51.655'	119°8.116'	1271
SOCAL_H_73_HS_C4	20 Dec 2021	22 May 2022	32°51.376'	119°8.337'	1246
SOCAL_H_73_HW_C4	20 Dec 2021	22 May 2022	32°51.696'	119°8.609'	1257
SOCAL_H_74_HE	23 May 2022	15 Oct 2022	32°51.654'	119°8.118'	1272
SOCAL_H_74_HS_C4	10 Jun 2022	15 Oct 2022	32°51.354'	119°8.363'	1244
SOCAL_H_74_HW_C4	10 Jun 2022	15 Oct 2022	32°51.673'	119°8.597'	1254
SOCAL_H_75_HE	16 Oct 2022	18 Apr 2023	32°51.730'	119°8.112'	1285
SOCAL_H_75_HS_C4	16 Oct 2022	17 Apr 2023	32°51.359'	119°8.354'	1248
SOCAL_H_75_HW_C4	17 Oct 2022	17 Apr 2023	32°51.678'	119°8.605'	1256
SOCAL_E_63_EE_C4	15 Mar 2018	05 Jul 2018	32°39.523'	119°28.627'	1326
SOCAL_E_63_ES	15 Mar 2018	11 Jul 2018	32°39.207'	119°29.073'	1329
SOCAL_E_63_EW_C4	15 Mar 2018	05 Jul 2018	32°39.388'	119°29.289'	1330
SOCAL_N_68_NE	29 Apr 2020	15 Oct 2020	32°22.185'	118°33.875'	1298
SOCAL_N_68_NS_C4	27 May 2020	15 Oct 2020	32°21.895'	118°34.105'	1338
SOCAL_N_68_NW_C4	27 May 2020	15 Oct 2020	32°22.185'	118°34.335'	1343

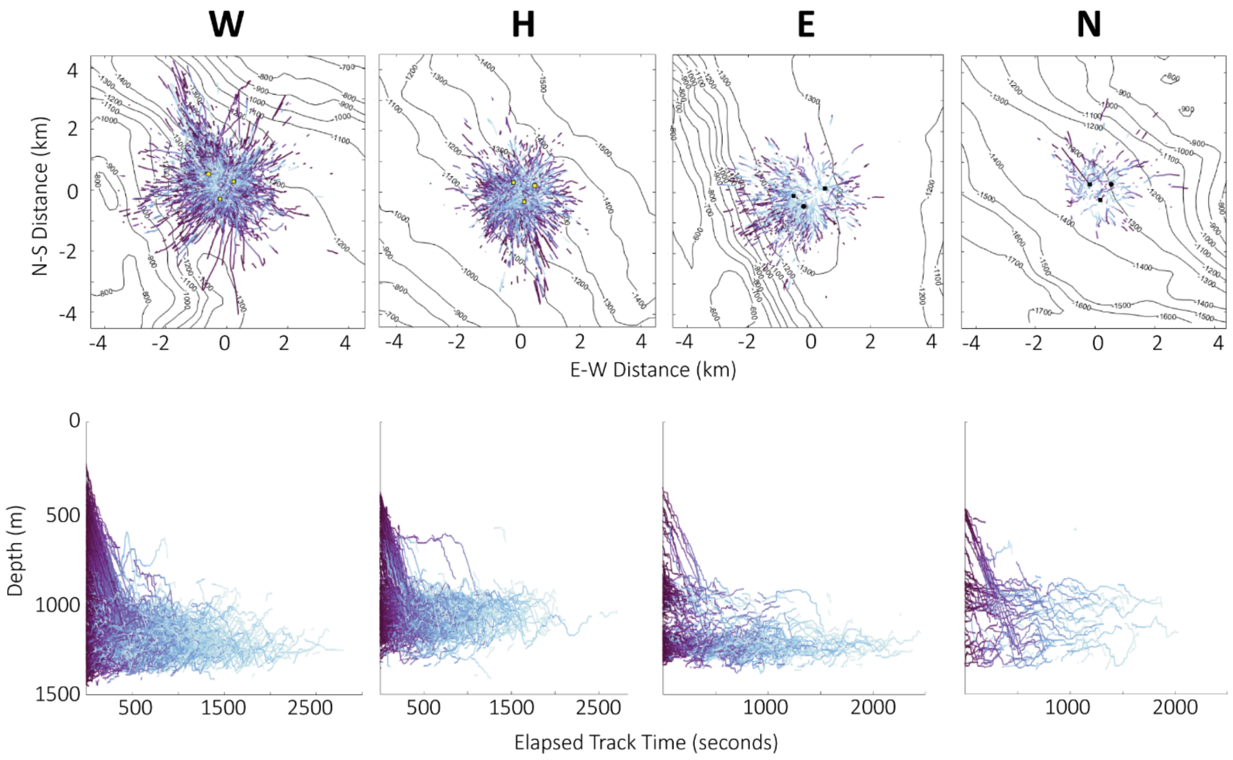

Supplemental Figure 1. Spatial view of all tracked whales per site. Columns represent site, and rows represent the XY view (top) and the Z view (bottom). Tracks are colored by normalized track time, with the start of the track in purple and the end of the track in blue. Contour lines every 100 meters indicate bathymetry, square markers represent 4-channel arrays and circle markers represent single-channel hydrophones. Black markers are used at sites with only one deployment, yellow markers are used at sites with multiple deployments. Yellow markers denote the array position of the first deployment; see also Supplemental Figure 3.

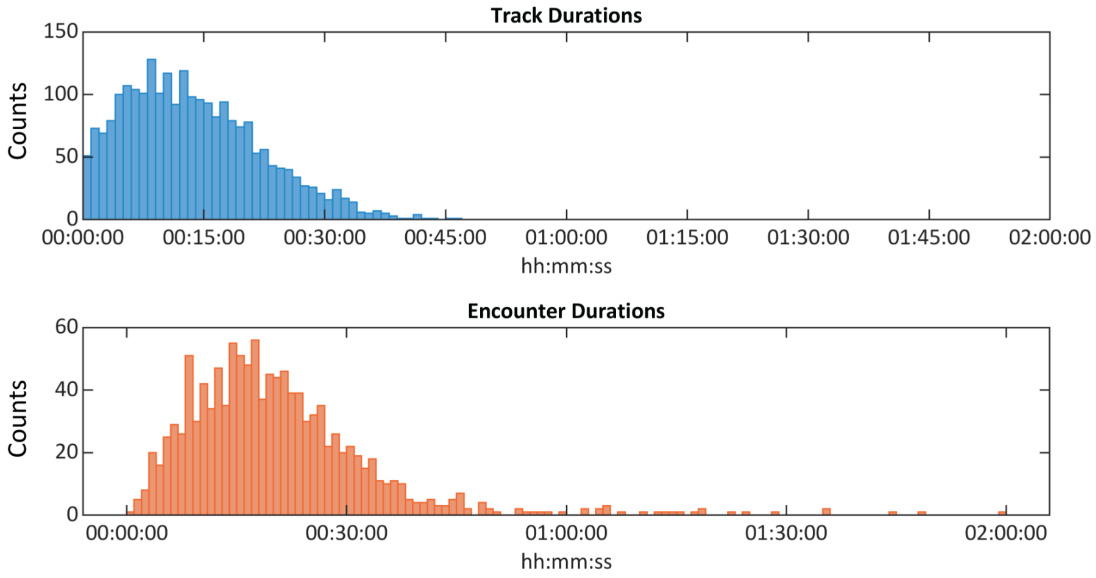

Supplemental Figure 2. Track and encounter duration histograms. Track durations represent the time that an individual goose-beaked whale was tracked, and encounter durations represent the time that an encounter of whales was tracked. Detections were considered as part of the same encounter if there were no gaps in detections exceeding 30 minutes.

Supplemental Table 2. Dunn post-hoc test p-values from pairwise comparisons for median distance above the seafloor per site.

Pairwise Comparison (site)	p-value
W – H	< 0.001
W – E	< 0.001
W – N	0.518
H – E	< 0.001
H – N	< 0.001
E – N	< 0.001

Supplemental Table 3. Dunn post-hoc test p-values from pairwise comparisons for median swim speeds at depth per site.

Pairwise Comparison (site)	p-value
W – H	< 0.001
W – E	< 0.001
W – N	0.175
H – E	0.219
H – N	0.561
E – N	0.143

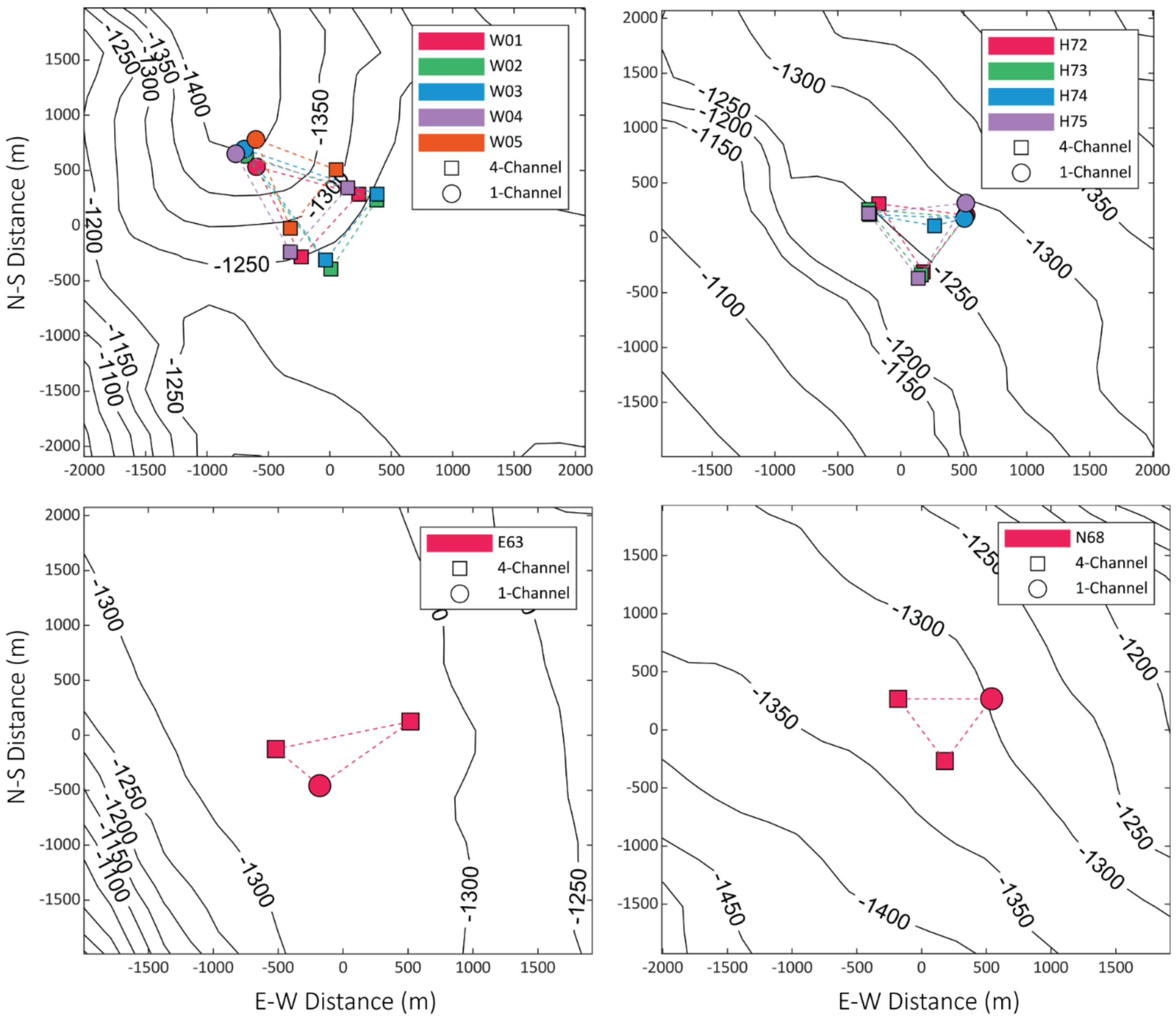

Supplemental Figure 3. Instrument positions for all deployments at all sites.

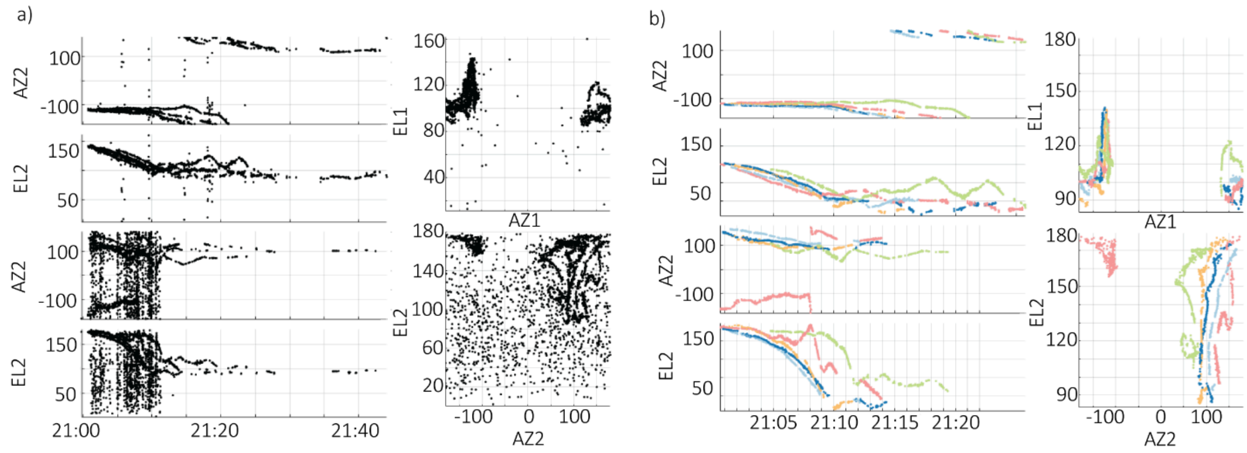

Supplemental Figure 4. Before (a) and after (b) track cleaning on the *brushDOA* interface in the *Where's Whaledo* software package (Snyder et al., 2024). Dots represent detections, black dots are unlabeled and colored dots have been assigned a whale number. This encounter was recorded at site W on September 5th, 2023.

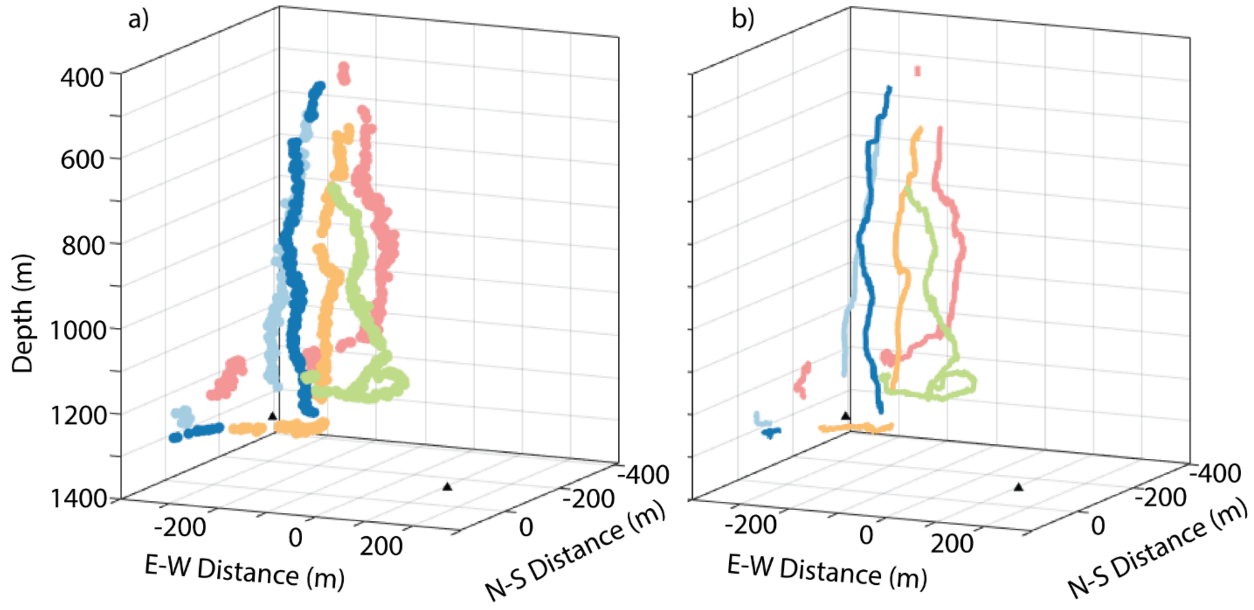

Supplemental Figure 5. Track smoothing results for 3D whale tracks. Points represent localized echolocation clicks, colors denote individuals (a). Lines represent smoothed whale locations from the Kalman filter approach, colors denote individuals (b). This encounter was recorded at site W on September 5th, 2023; see Supplemental Figure 4 for the azimuth-elevation view of this encounter.

a)

```
bestModel = gam(groupSize~
                + s(dfMod$jd,bs='cc')
                + s(presBins,bs='ts'),
              data=dfMod,family=tw(),method='REML')
summary(bestModel)
# Family: Tweedie(p=1.99)
# Link function: log
#
# Formula:
# groupSize ~ +s(dfMod$jd, bs = "cc") + s(presBins, bs = "ts")
#
# Parametric coefficients:
#             Estimate Std. Error t value Pr(>|t|)
# (Intercept)  1.27276    0.01782   71.43   <2e-16 ***
# ---
# Signif. codes:  0 '***' 0.001 '**' 0.01 '*' 0.05 '.' 0.1 ' ' 1
#
# Approximate significance of smooth terms:
#               edf Ref.df      F p-value
# s(dfMod$jd) 2.189      8  1.053 0.00874 **
# s(presBins) 2.517      9 14.261 < 2e-16 ***
# ---
# Signif. codes:  0 '***' 0.001 '**' 0.01 '*' 0.05 '.' 0.1 ' ' 1
#
# R-sq.(adj) =  0.0827   Deviance explained = 9.77%
# -REML = 2508.1  Scale est. = 0.38752   n = 1205
```

b)

```
bestModel = gam(groupSize~
                + s(dfMod$jd,bs='cc')
                + s(dfMod$dayNight,bs='cc')
                + presBins,
              data=dfMod,family=tw(),method='REML')
summary(bestModel)
# Family: Tweedie(p=1.99)
# Link function: log
#
# Formula:
# groupSize ~ +s(dfMod$jd, bs = "cc") + s(dfMod$dayNight, bs = "cc") +
#     presBins
#
# Parametric coefficients:
#             Estimate Std. Error t value Pr(>|t|)
# (Intercept) 0.822161   0.034260  23.998   <2e-16 ***
# presBins    0.002949   0.001441   2.046   0.0412 *
# ---
# Signif. codes:  0 '***' 0.001 '**' 0.01 '*' 0.05 '.' 0.1 ' ' 1
#
# Approximate significance of smooth terms:
#                     edf Ref.df     F  p-value
# s(dfMod$jd)       5.323      8 5.183  < 2e-16 ***
# s(dfMod$dayNight) 2.792      8 2.514 3.27e-05 ***
# ---
# Signif. codes:  0 '***' 0.001 '**' 0.01 '*' 0.05 '.' 0.1 ' ' 1
#
# R-sq.(adj) =  0.0768   Deviance explained = 10.1%
# -REML = 1018.2  Scale est. = 0.35758   n = 607
```

Supplemental Figure 6. Final GAM model equations and model summary for site W (a) and site H (b).

Supplemental Table 4. Parameters used in Monte-Carlo simulations for detection probability.

Modeled Parameter	Modeled Values
Dive altitude above seafloor (m)	275, 300, 325
Source level (peak-to-peak, dB re 1 μPa^2)	221, 222, 223
Mean descent angle	60°
Depth of clicking start (m)	200
Directivity	23, 24, 25

Supplemental GIFs. Tracking results for a few encounters per site. Individual whales are denoted by separate colors. Solid lines indicate tracked positions, dashed lines indicate data gaps. Black or white squares denote the position of the 4-hydrophone arrays. Gray gradient surface represents bathymetry.
