# Supplementary figures and images for: Long-term monitoring of Ziphius cavirostris behavior using 3D tracking from fixed hydrophone arrays off Southern California
Source: Sci Rep. 2025 Nov 19;15:40859. doi: 10.1038/s41598-025-24490-x (PMC12630762; doi:10.1038/s41598-025-24490-x)

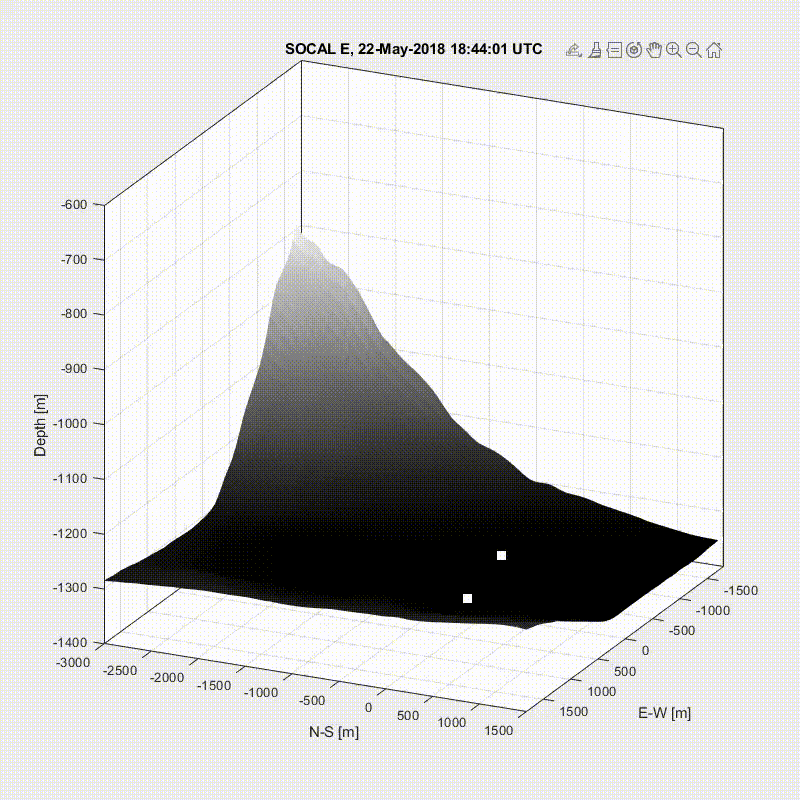

Supplement: Supplementary file 2 — Supplementary Information 2. [file 41598_2025_24490_MOESM2_ESM.zip › supplemental_GIFs/coordinated_behaviors/SOCAL_E_63_track278.gif]

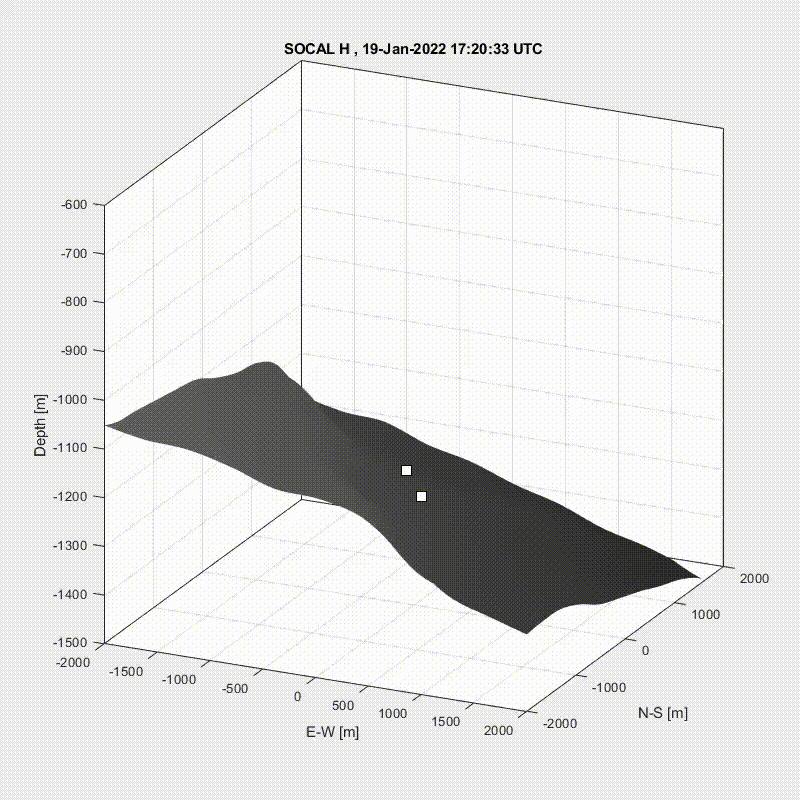

Supplement: Supplementary file 2 — Supplementary Information 2. [file 41598_2025_24490_MOESM2_ESM.zip › supplemental_GIFs/coordinated_behaviors/SOCAL_H_73_track33.gif]

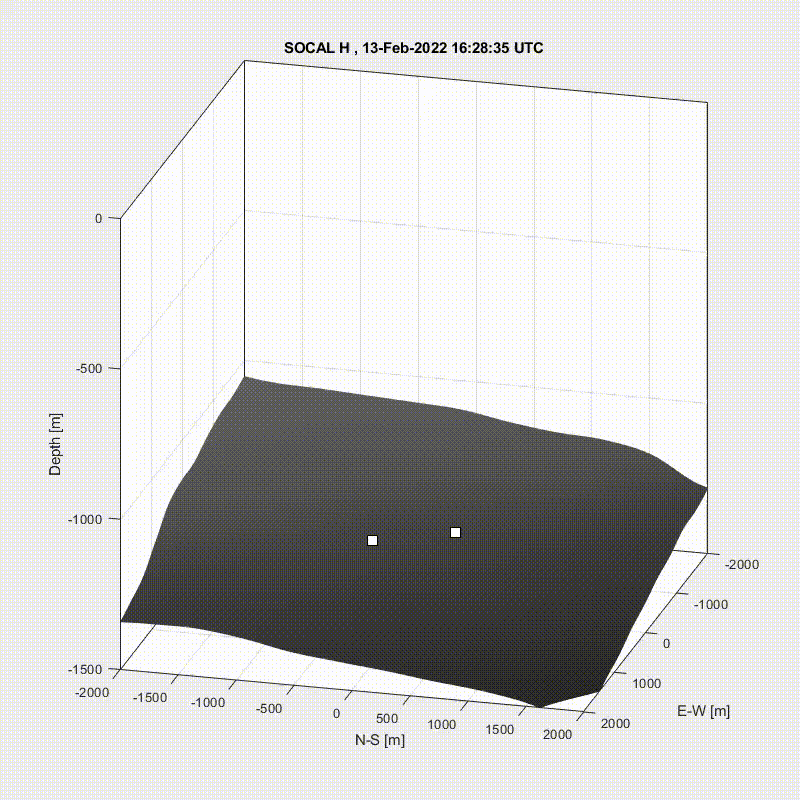

Supplement: Supplementary file 2 — Supplementary Information 2. [file 41598_2025_24490_MOESM2_ESM.zip › supplemental_GIFs/coordinated_behaviors/SOCAL_H_73_track72.gif]

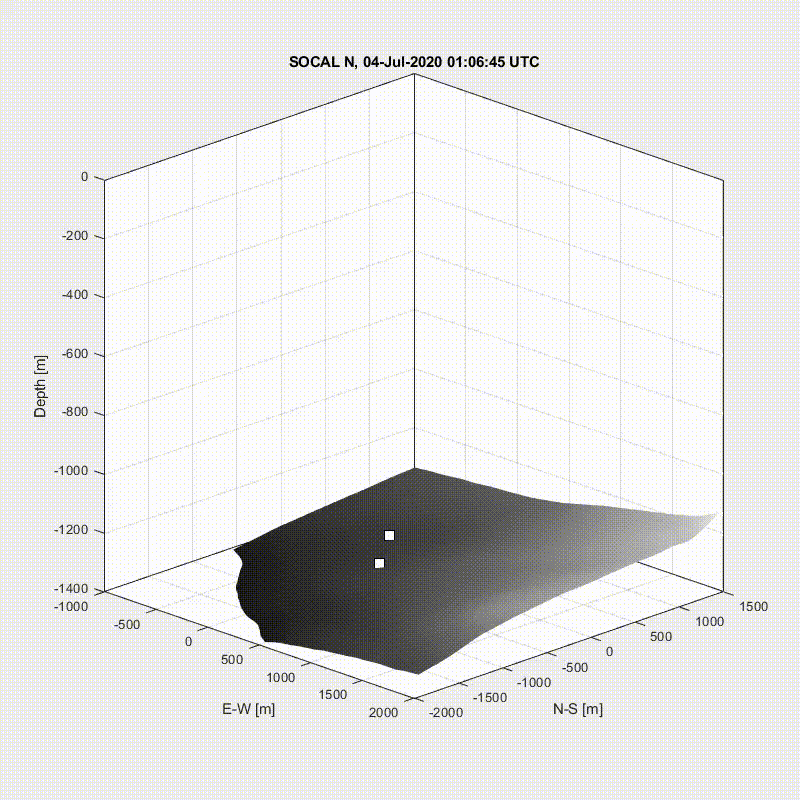

Supplement: Supplementary file 2 — Supplementary Information 2. [file 41598_2025_24490_MOESM2_ESM.zip › supplemental_GIFs/coordinated_behaviors/SOCAL_N_68_track40.gif]

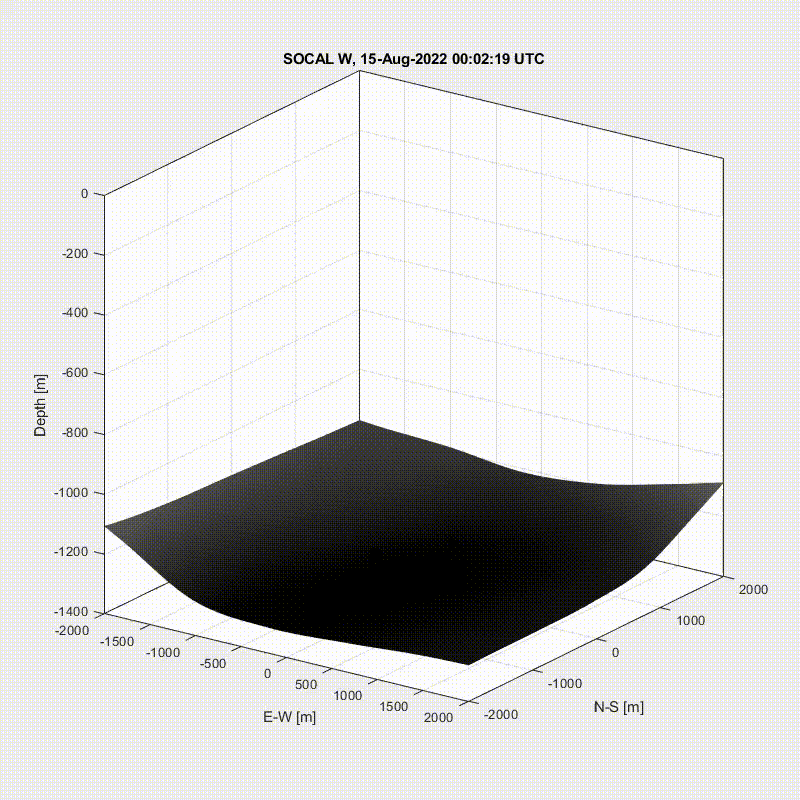

Supplement: Supplementary file 2 — Supplementary Information 2. [file 41598_2025_24490_MOESM2_ESM.zip › supplemental_GIFs/coordinated_behaviors/SOCAL_W_03_track423.gif]

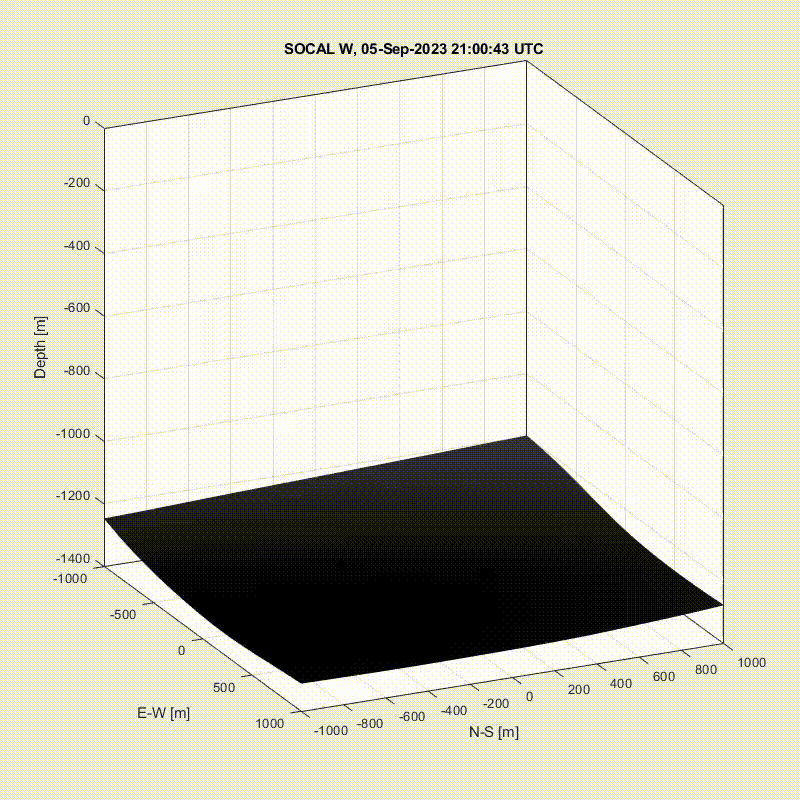

Supplement: Supplementary file 2 — Supplementary Information 2. [file 41598_2025_24490_MOESM2_ESM.zip › supplemental_GIFs/coordinated_behaviors/SOCAL_W_05_track853.gif]

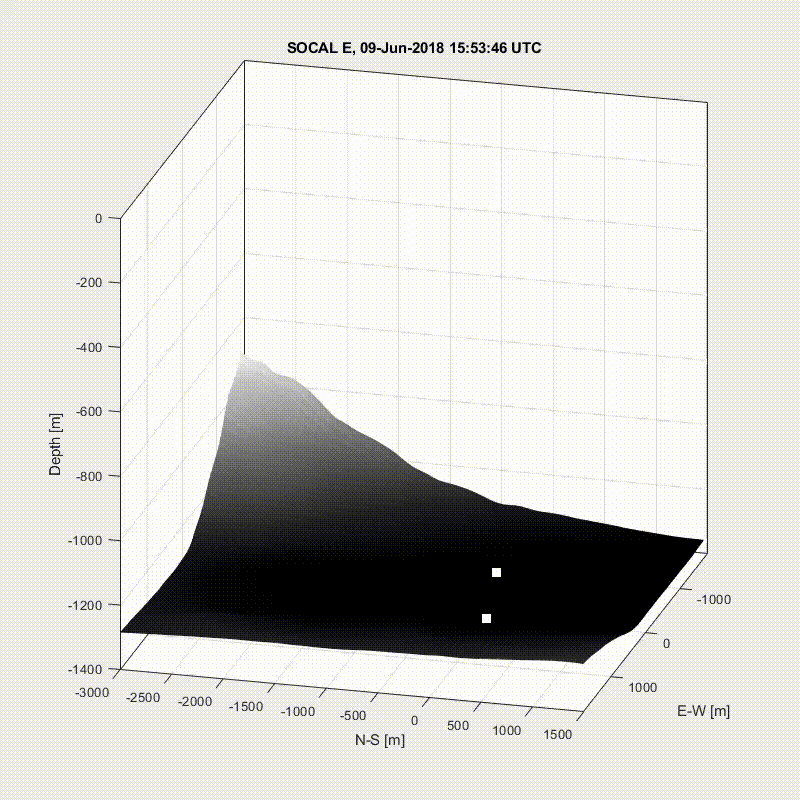

Supplement: Supplementary file 2 — Supplementary Information 2. [file 41598_2025_24490_MOESM2_ESM.zip › supplemental_GIFs/general_examples/SOCAL_E_63_track376.gif]

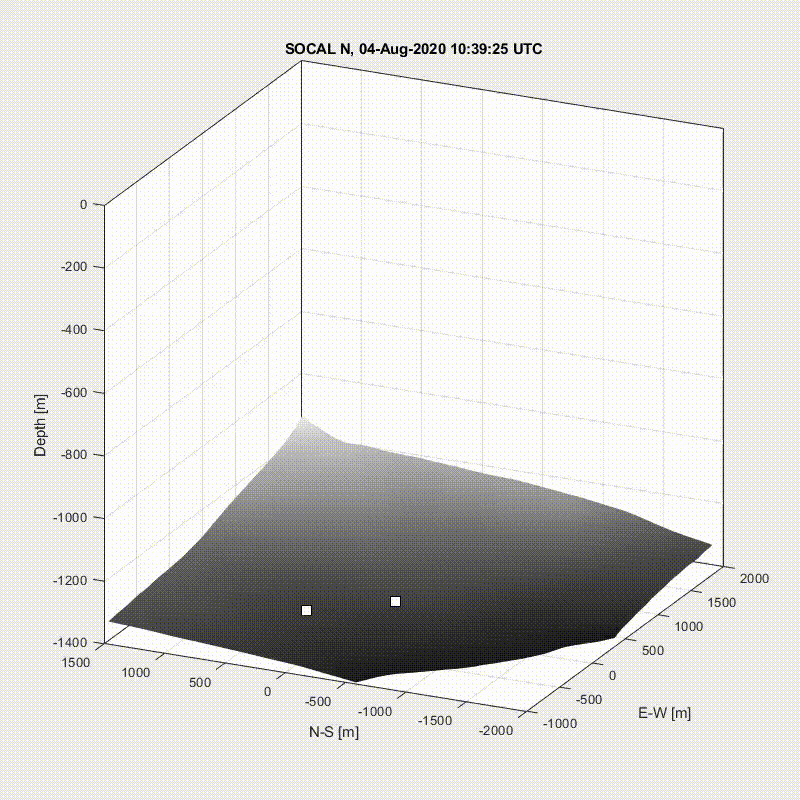

Supplement: Supplementary file 2 — Supplementary Information 2. [file 41598_2025_24490_MOESM2_ESM.zip › supplemental_GIFs/general_examples/SOCAL_N_68_track74.gif]

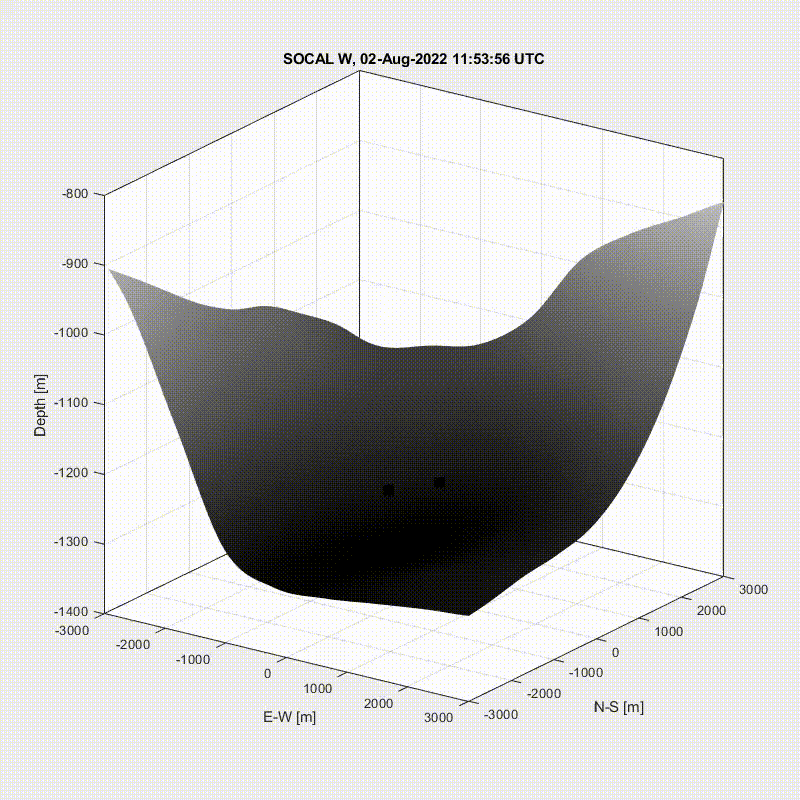

Supplement: Supplementary file 2 — Supplementary Information 2. [file 41598_2025_24490_MOESM2_ESM.zip › supplemental_GIFs/general_examples/SOCAL_W_03_track339.gif]
